# Supplementary figures and images for: A Rab1 interactome illuminates a dual role in autophagy and membrane trafficking
Source: J Cell Biol. 2026 Jan 6;225(3):e202507084. doi: 10.1083/jcb.202507084 (PMC12772502; doi:10.1083/jcb.202507084)

E

Immunoblot: RABEP1

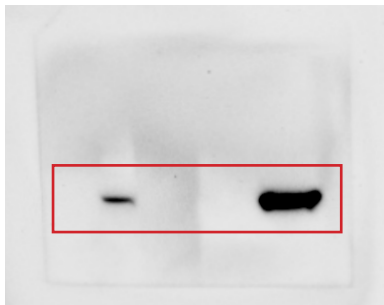

Immunoblot: PPP1R37

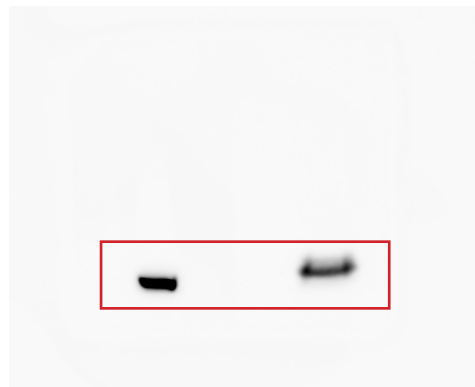

Immunoblot: CLEC16A

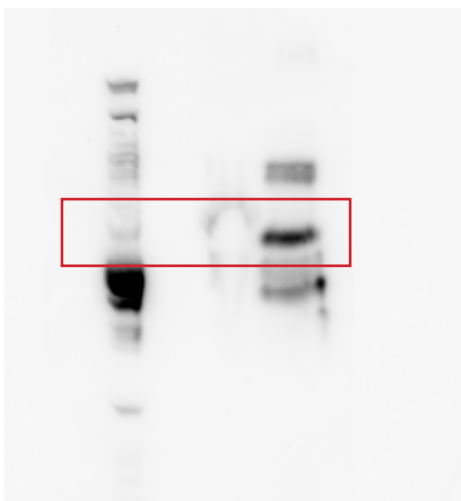

Immunoblot: Rab1A

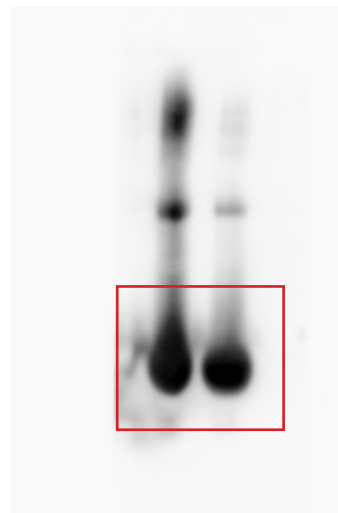

Supplement: SourceData F1 — is the source file for Fig. 1. [file jcb_202507084_sourcedataf1.pdf]

C

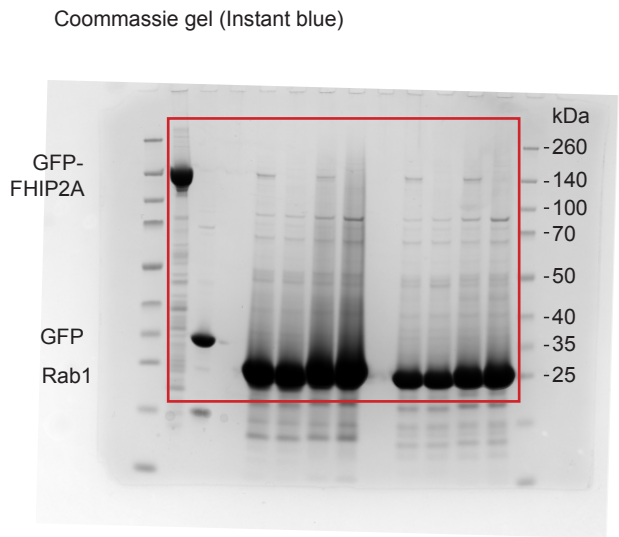

F

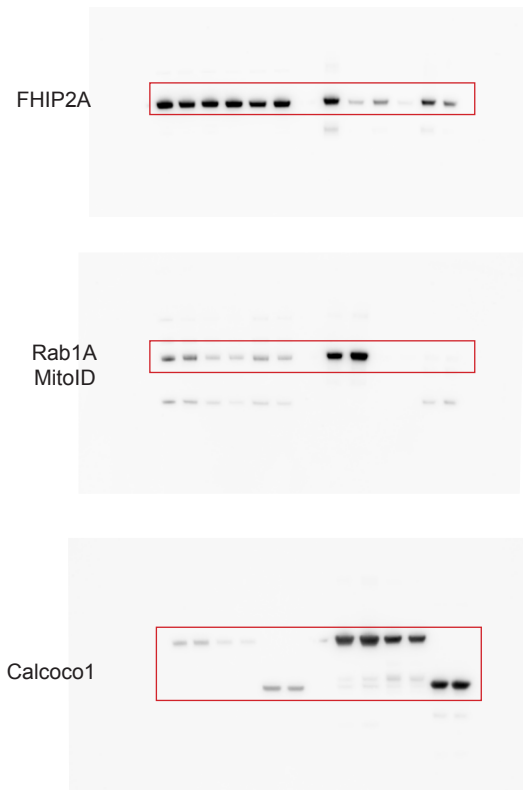

Supplement: SourceData F2 — is the source file for Fig. 2. [file jcb_202507084_sourcedataf2.pdf]

A

Coommassie gel (Instant blue)

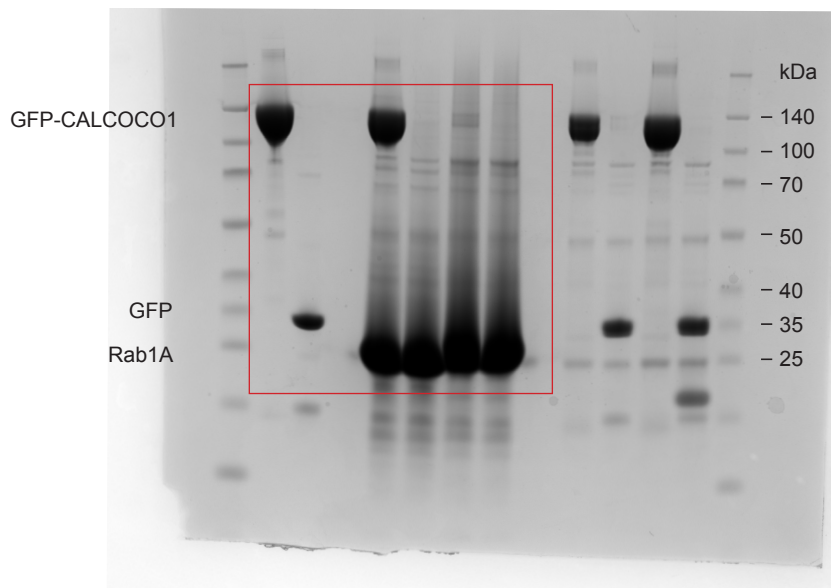

B

Coommassie gel (Instant blue)

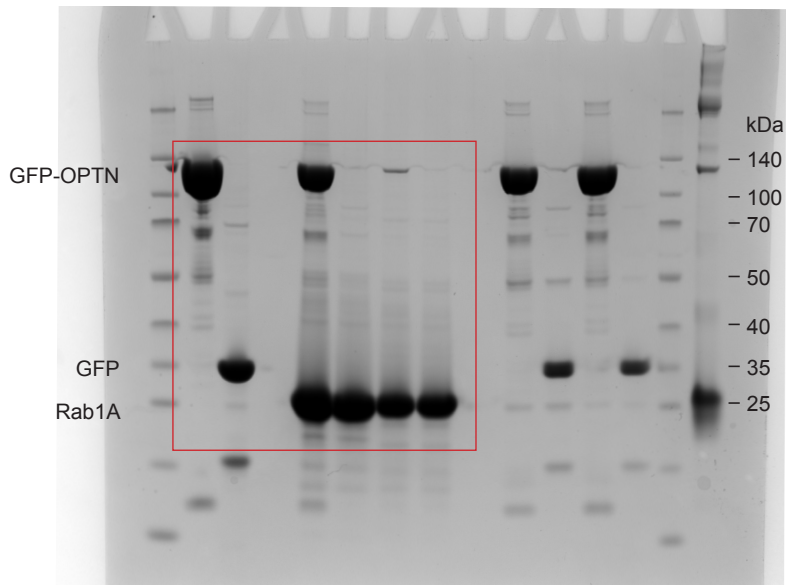

Supplement: SourceData F3 — is the source file for Fig. 3. [file jcb_202507084_sourcedataf3.pdf]

B

Immunoblot: HA (Rab1 MitolD)

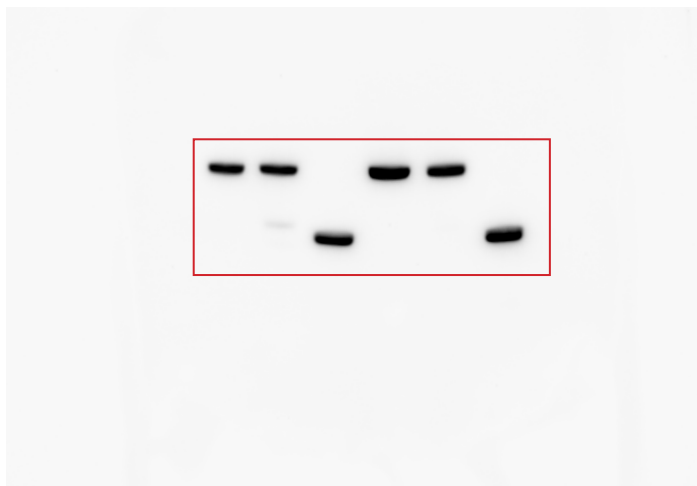

Immunoblot: Tubulin

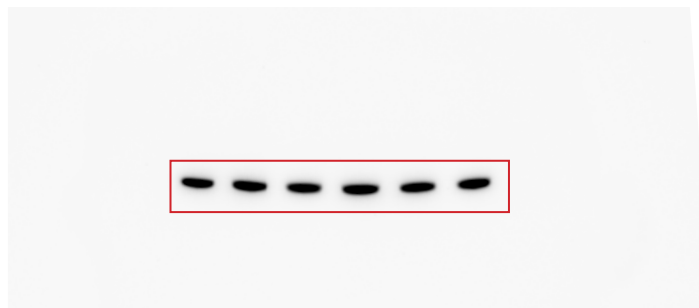

Supplement: SourceData FS1 — is the source file for Fig. S1. [file jcb_202507084_sourcedatafs1.pdf]

A

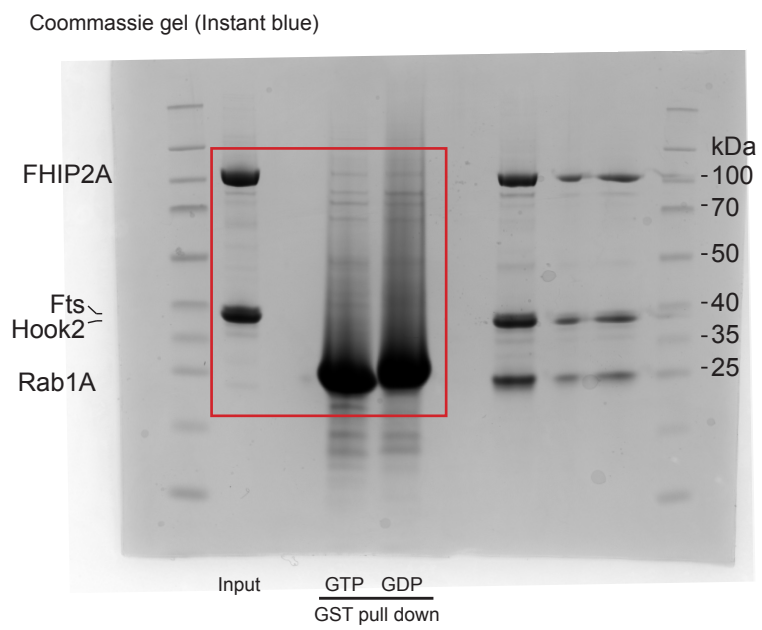

B

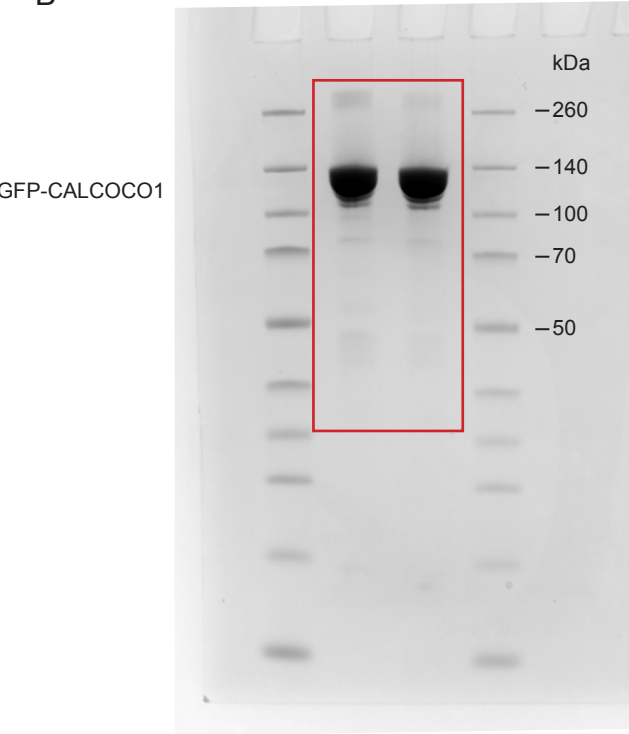

E

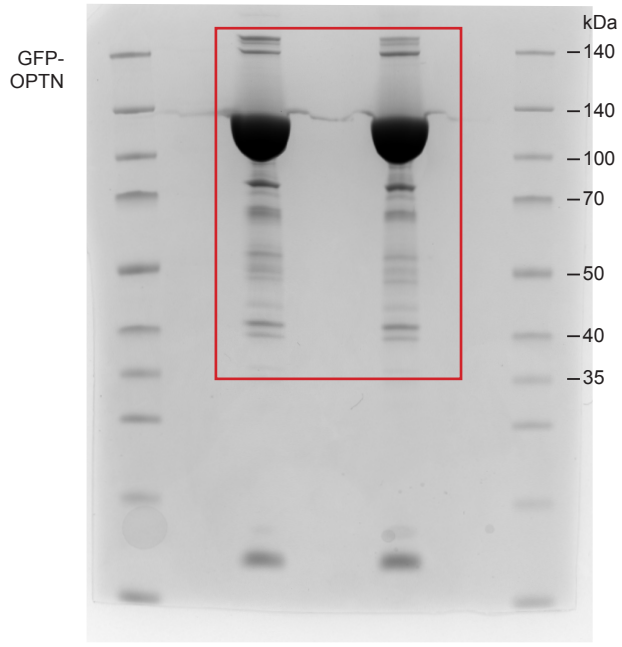

Supplement: SourceData FS2 — is the source file for Fig. S2. [file jcb_202507084_sourcedatafs2.pdf]

C

OPTN

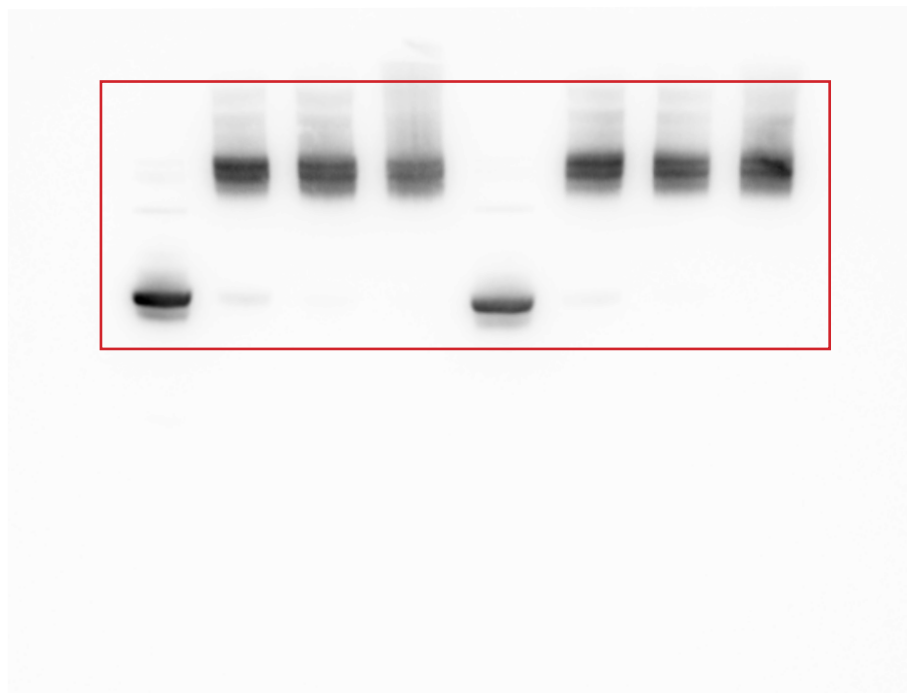

GAPDH

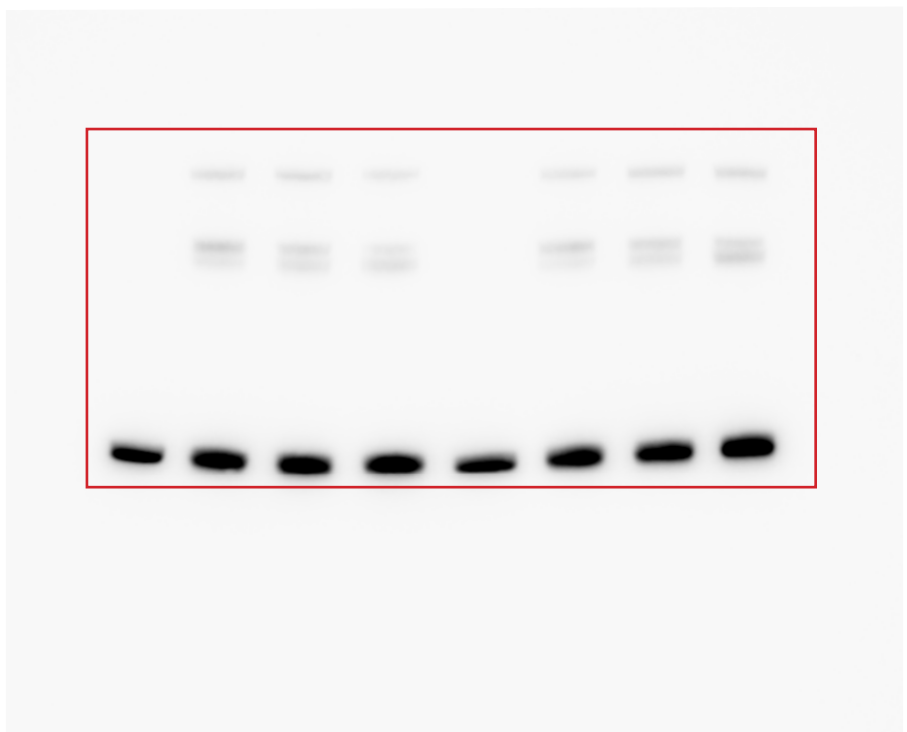

Supplement: SourceData FS3 — is the source file for Fig. S3. [file jcb_202507084_sourcedatafs3.pdf]
