# Supplementary material for: A Rab1 interactome illuminates a dual role in autophagy and membrane trafficking
Source: J Cell Biol. 2026 Jan 6;225(3):e202507084. doi: 10.1083/jcb.202507084 (PMC12772502; doi:10.1083/jcb.202507084)

Source Data: Figure 5

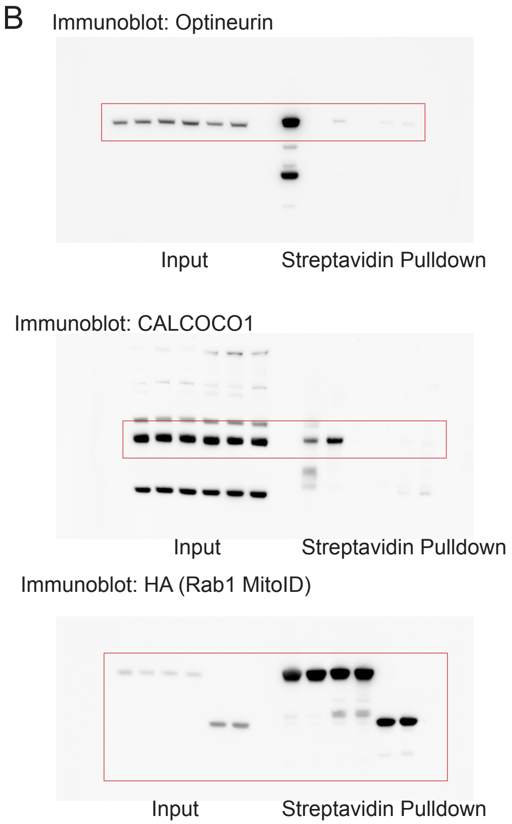

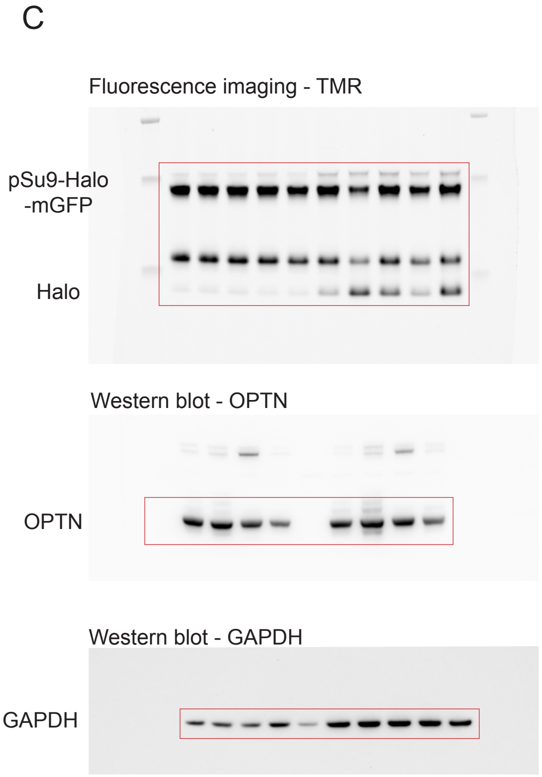

Supplement: SourceData F5 — is the source file for Fig. 5. [file jcb_202507084_sourcedataf5.pdf]
